# Interactive effect of elevated CO_2_ and drought on physiological traits of *Datura stramonium*

**DOI:** 10.3389/fpls.2022.929378

**Published:** 2022-10-26

**Authors:** Muhammad Mansoor Javaid, Singarayer Florentine, Athar Mahmood, Allah Wasaya, Talha Javed, Abdul Sattar, Naeem Sarwar, Hazem M. Kalaji, Hafiz Bashir Ahmad, Jacek Worbel, Mohammed A. A. Ahmed, Arkadiusz Telesiński, Jacek Mojski

**Affiliations:** ^1^ Department of Agronomy, College of Agriculture, University of Sargodha, Sargodha, Pakistan; ^2^ Future Regions Research Centre, Federation University Australia, Mount Helen, VIC, Australia; ^3^ Department of Agronomy, University of Agriculture Faisalabad, Faisalabad, Pakistan; ^4^ College of Agriculture, BZU, Bahadur Sub Campus, Layyah, Pakistan; ^5^ College of Agriculture, Fujian Agriculture and Forestry University, Fuzhou, China; ^6^ Department of Agronomy, Bahauddin Zakariya University, Multan, Pakistan; ^7^ Department of Plant Physiology, Institute of Biology, Warsaw University of Life Sciences SGGW, Warsaw, Poland; ^8^ Institute of Technology and Life Sciences, National Research Institute, Raszyn, Poland; ^9^ Department of Forestry, College of Agriculture, University of Sargodha, Sargodha, Pakistan; ^10^ Department of Bioenegineering, West Pomerania, University of Technology Szczecin, Szczecin, Poland; ^11^ Plant Production Department (Horticulture-Medicinal and Aromatic Plants), Faculty of Agriculture (Saba Basha), Alexandria University, Alexandria, Egypt; ^12^ Twój Swiat Jacek Mojski, Lukow, Poland; ^13^ Fundacja Zielona Infrastruktura, Lukow, Poland

**Keywords:** gas exchange, photosystem II activity, fluorescence, water use efficiency, electron transport rate

## Abstract

Rising atmospheric CO_2_ concentrations are known to influence the response of many plants under drought. This paper aimed to measure the leaf gas exchange, water use efficiency, carboxylation efficiency, and photosystem II (PS II) activity of *Datura stramonium* under progressive drought conditions, along with ambient conditions of 400 ppm (aCO_2_) and elevated conditions of 700 ppm (eCO_2_). Plants of *D. stramonium* were grown at 400 ppm and 700 ppm under 100 and 60% field capacity in a laboratory growth chamber. For 10 days at two-day intervals, photosynthesis rate, stomatal conductance, transpiration rate, intercellular CO_2_ concentration, water use efficiency, intrinsic water use efficiency, instantaneous carboxylation efficiency, PSII activity, electron transport rate, and photochemical quenching were measured. While drought stress had generally negative effects on the aforementioned physiological traits of *D. stramonium*, it was found that eCO_2_ concentration mitigated the adverse effects of drought and most of the physiological parameters were sustained with increasing drought duration when compared to that with aCO_2_. *D. stramonium*, which was grown under drought conditions, was re-watered on day 8 and indicated a partial recovery in all the parameters except maximum fluorescence, with this recovery being higher with eCO_2_ compared to aCO_2_. These results suggest that elevated CO_2_ mitigates the adverse growth effects of drought, thereby enhancing the adaptive mechanism of this weed by improving its water use efficiency. It is concluded that this weed has the potential to take advantage of climate change by increasing its competitiveness with other plants in drought-prone areas, suggesting that it could expand into new localities.

## Introduction

Since the Industrial Revolution, atmospheric CO_2_ has been increasing and is predicted to reach 800 ppm at the end of this century (Valone, 2021). Elevated CO_2_ (eCO_2_) stimulates global warming, causing rapid changes in the climate of the earth, affecting variations in temperature, precipitation amounts and intensity patterns (Skendžić et al., 2021). Moreover, it has been reported that global warming acts to cause deficits in atmospheric vapor pressure, resulting in drought stress in plants (Song et al., 2021). This rapid climate change may also alter the geographical ranges of species (Skendžić et al., 2021), and it is, therefore, predicted that species that can tolerate warmer and drier weather will have competitive benefits over non-tolerant species (Karimizadeh et al., 2021). Changes in precipitation patterns will also affect the success of species invasion and the nature of ecosystems in general. It has been reported that crop species under eCO_2_ act to enhance photosynthesis when other factors like light, water, and nutrients are not limiting (Boucher et al., 2009; AbdElgawad et al., 2015; AbdElgawad et al., 2016; Cruz et al., 2018; Alba et al., 2019). Dusenge et al. (2019) concluded that the current ambient CO_2_ concentration is a limiting factor to growth, so an increase in CO_2_ concentration clearly has the potential to increase the growth and yield of plants.

Elevated CO_2_ increases the activity rate of Rubisco, which results in increased plant growth (López-Calcagno et al., 2020). This will be beneficial for agriculture because of greater crop productivity, but the growth of weeds, especially invasive species, will also be enhanced (Wu et al., 2017). Physiologically, elevated CO_2_ increases biomass production by reducing water loss through the stomata, in conjunction with an increase in root growth and improved root structure (Li et al., 2020). Stomatal conductance is based on CO_2_, which reduces the transpiration rate of crop species grown under elevated CO_2_ with ample water supplies (Kimball, 2016). However, the response of stomata to eCO_2_ is dependent on the interactive effect of elevated CO_2_ and soil water content (Xu et al., 2016). It is well documented that an increase in CO_2_ concentration causes partial stomatal closure, which reduces leaf transpiration and increases net carbon assimilation (Dai, 2013; AbdElgawad et al., 2015; Cruz et al., 2018).

Elevated CO_2_ increases the net assimilation rate by up to 30%, which can lead to a significant increase in dry matter and yield of crops (Robredo et al., 2007; Cruz et al., 2018; Pan et al., 2018). Wheat crops grown under 550 ppm CO_2_ concentration have been shown to increase their yield by 15%, the canopy temperature by 0.85°C, and have reduced their evapotranspiration by 13%, consequently increasing the water use efficiency by 18%. Rising levels of CO_2_ have, therefore, potential beneficial effects on the growth and yield of plants, especially in areas where drought causes crop failure (Jin et al., 2019). According to AbdElgawad et al. (2016), eCO_2_ has been shown to mitigate the stress impact of drought, and they quoted examples of barley and alfalfa crop growth reduction under drought stress (Zeid and Shedeed, 2006; Robredo et al., 2007). However, despite these beneficial effects on crops, the rising level of atmospheric CO_2_ may have negative consequences for yield losses due to weed-crop competition (Ziska et al., 2019). Observations have shown that while vegetative growth of C_3_ crops is favored over C_4_ weeds under elevated CO_2_ (Ziska and Caulfield, 2000), C_3_ weeds may be favored over C_3_ crops (Ziska et al., 2019). It has recently been suggested that the effect of drought may be overcome by some compensatory mechanism within the plants (Qi et al., 2021). Numerous reports showed that plants that were grown under eCO_2_ dried more slowly as water was withheld due to lower stomatal conductance and transpiration rate (Yan et al., 2017; Dikšaitytė et al., 2019; Pastore et al., 2020). It is postulated that the benefit gained by weeds from eCO_2_ under drought conditions is linked to physiological traits like chlorophyll content, gas exchange, water use efficiency, and PSII activity of plants (Li et al., 2019). Elevated CO_2_ increased the growth and reproduction of both indigenous and non-indigenous weeds, which has resulted in an enhancement of their competitive ability. This outcome supports the hypothesis of Blossey and Notzold (1995), who hypothesized that there is an evolution of increased competitive ability in invasive plants. It is well documented that invasive species affect the growth, development, and reproduction of native species by altering the ecosystem and introducing direct competition for essential resources (Werner et al., 2008). Although gaining an understanding of the effect of increased atmospheric CO_2_ combined with drought on the growth and yield of crops has a priority over weed studies (Nguyen et al., 2017), weed infestation is, nevertheless, an important impediment to crop productivity and needs to be investigated in its own right.

*Datura stramonium*, commonly known as thorn apple or Jimson weed, is an invasive C_3_ weed species belonging to the family Solanaceae (Chadha et al., 2020). It is grown in subtropical and temperate regions worldwide (Chadha et al., 2020). It has fast seedling growth with a short vegetative stage and is characterized by indeterminate growth habits. Broad leaves and sympodial branches allow this weed to shade the surrounding areas and increase its competitive ability. It is a common weed found in soybean, potato, tomato, and tobacco crops. *D. stramonium* has narcotic properties and contains tropane alkaloids, mainly scopolamine, hyoscyamine, and atropine chemicals, which are known to be poisonous for humans, cattle, and horses (Baloch et al., 2017). These characters restrict the agricultural use of this species, but some studies have shown the potential of *D. stramonium* for bio-oil production (Aysu and Durak, 2015; Durak and Aysu, 2016). Therefore, it is important to develop a good understanding of how *D. stramonium* is likely to respond to changing environments, indicating that the lack of scientific literature regarding the effect of eCO_2_ concentration on the physiological processes of *D. stramonium* is a significant problem. The current study focuses on an understanding of the regulation of gas exchange, photosynthetic efficiency of PSII, and water use efficiency of *D. stramonium* at 400 and 700 ppm CO_2_ grown under normal irrigation and drought conditions, and reflects on the implications of these results for their growth and invasive characteristics.

## Materials and methods

### Seed collection

Seeds of *D. stramonium* were obtained by removing the heads from mature plants, and subsequently drying and threshing them to remove extraneous material. These heads were collected from more than 100 different plants in Ballarat, Victoria, Australia. The cleaned seeds were stored in a dried amber glass bottle at 19°C in the seed ecology laboratory of Federation University, Mt. Helen, Australia, until used in the experiment.

### Condition of the experiment

Experiments were conducted at Federation University (37°37.39°S, 143°53.27°E) in two CO_2_ chambers (2.1 m length, 2.1 width, and 2.0 m height) (Steridium Pty. Ltd., Brendale, Qld, Australia). One CO_2_ chamber was set at 400 ppm CO_2_ concentration (aCO_2_), while the other was set at 700 ppm CO_2_ concentration (eCO_2_). The average chamber temperature was maintained at 22/18°C day/night alternating temperature with 60% humidity. Twenty plastic pots (13 cm wide and 14 cm high) were filled, each with 1.2 kg of a 2:1 mixture of garden soil and a commercially available potting mixture. Three seeds of *D. stramonium* were sown in the center of each pot. Out of the twenty pots, 10 randomly selected samples were kept in the cabinet maintained at an ambient CO_2_ level (400 ppm), and the remaining 10 pots were kept in the second cabinet maintained at an elevated CO_2_ level (700 ppm). The pots were watered daily, and seedlings were thinned at the four-leaf stage, leaving one seedling in each pot. Two moisture levels (well-watered and drought) were also maintained in each CO_2_ chamber. The well-watered treatments were maintained at 100% field capacity and drought treatments at 60% field capacity. Drought treatment commenced 25 days after sowing. These water regimes were selected as different plant species showed variable responses to eCO_2_ concentrations under drought conditions. Water holding capacity was determined according to Bajwa et al. (2019). Briefly, 10 kg of the soil was placed into three pots and saturated with tap water. The pot surface was covered with black plastic, and the pots were allowed to drain for 48 h to determine the water hole. After this time, the plastic sheet was removed and three soil samples (each weighing 300 g) were taken from the mid position of each pot. These samples were weighed (wet weight of soil, A) before being oven-dried (90°C for 72 h) and reweighed (dry weight of soil, B). The field capacity was then calculated by the formula (A − B) × 100/B. The 60% field capacity was determined based on that fraction of the water holding capacity.

Of the 10 pots in each chamber, half were subjected to well-watered conditions and the remaining half were subjected to a drought regime. The pots were weighed to maintain an accurate amount of water field capacity levels, noting that the weight of the growing plant was much smaller than that of the soil in the pot. There were five replications (one pot for each replication) for each treatment and each replication consisted of one plant. In drought treatments, water was withheld until Day 8 in both CO_2_ chambers, after which drought treatment pots were re-watered to investigate the recovery response of the *D. stramonium* plants. The amount of water added was calculated based on pot weight, and 60% field capacity was maintained till Day 10. In the well-watered treatments, water was added on alternate days.

### Gas exchange and fluoresence

To evaluate the effect of CO_2_ and drought on physiological parameters, the LI-COR portable infrared CO_2_ gas analyzer was used (LI-6400 XT portable photosynthesis system, LI-COR, Biosciences, Lincoln, Nebraska, USA). Measurements were recorded with the following adjustments by LI-COR: The block temperature was set at 20°C, the photosynthetic photon flux density (PPFD) was 1,000 µmol m^−2^ s^−1^ with a red-blue light source, the leaf cuvette area was set at 2 cm^2^ and the flow rate was adjusted to 500 µmol m^−2^ s^−1^. The light conditions in the chamber were set for 12 h and 12 h dark. The mean value of VPD in the leaf cuvette was 1.75 ± 0.03 kPa. The CO_2_ concentration in the chamber was noted before each measurement, and adjusted to within ±10 ppm of the stated level. On alternate days, net photosynthesis rate, stomatal conductance, transpiration rate, and intercellular CO_2_ concentration were measured and the parameters were started to measure at 9:30 am. Water use efficiency was calculated by dividing the net photosynthesis rate with the transpiration rate; intrinsic water use efficiency was calculated by dividing the net photosynthesis rate with stomatal conductance; and instantaneous carboxylation efficiency was calculated by dividing the net photosynthesis rate by the intercellular CO_2_ concentration. Photosystem II (PSII) activity, such as minimum fluorescence, maximum fluorescence, the effective quantum efficiency of PSII, photochemical efficiency of PSII, photochemical quenching, and photosynthetic electron transport rate, were calculated according to the methods described by Maxwell and Johnson (2000). Maximum fluorescence was determined by applying a saturating light pulse of 7,000 µmol m^−2^s^−1^. The effective quantum efficiency of PSII was measured under actinic light of 180 µmol m^−2^s^−1^.

The effective quantum efficiency of PSII was calculated as


(1) $$\Phi_{PSII}=\frac{\left(F_{m}^{'}{\;}_{-}F_{s}\right)}{F_{m}^{'}}$$

Where Φ_PSII_ is the effective quantum efficiency of PSII, ${\text{F}}_{\text{m}}^{'}$ is the maximum fluorescence and F_s_ is the steady state fluorescence prior to the flash.

Photochemical efficiency of PSII ( ${\text{F}}_{\text{v}}^{'}/{\text{F}}_{\text{m}}^{'}$ ) was determined in terms of efficiency of energy harvesting by oxidized PSII using the following equation


(2) $$\frac{\overset{'}{F_{v}}}{\overset{'}{F_{m}}}=\frac{\overset{'}{F_{m}}-\overset{'}{F_{0}}}{\overset{'}{F_{m}}}$$

Where ${\text{F}}_{\text{m}}^{'}$ is the maximum fluorescence and $\overset{'}{F_{0}}$ is the minimum fluorescence.

The photochemical quenching was computed from


(3) $$qp=\frac{\overset{'}{F_{m}}-F_{s}}{\overset{'}{F_{m}}-\overset{'}{F_{o}}}$$

Where Fs is the steady-state fluorescence and $\overset{'}{F_{0}}$ is the minimum fluorescence.

### Statistical analysis

Data for gas exchange parameters were presented in graphs along with the standard error of each mean, and the graphs were prepared using the SigmaPlot 11 software. Regression analysis was performed on the data of photosynthesis rate, stomatal conductance, and transpiration rate under 8 days of drought conditions. To investigate the effects of time of observation (time), water conditions (water), and CO_2_ levels (CO_2_), physiological parameters were analyzed with Statisix 8.1 using three-factor ANOVA. All the main effects and two- and three-way interactions were examined using Tukey’s HSD test at a 5% probability level.

## Results

### Gas exchange parameters

**Figure 1:**
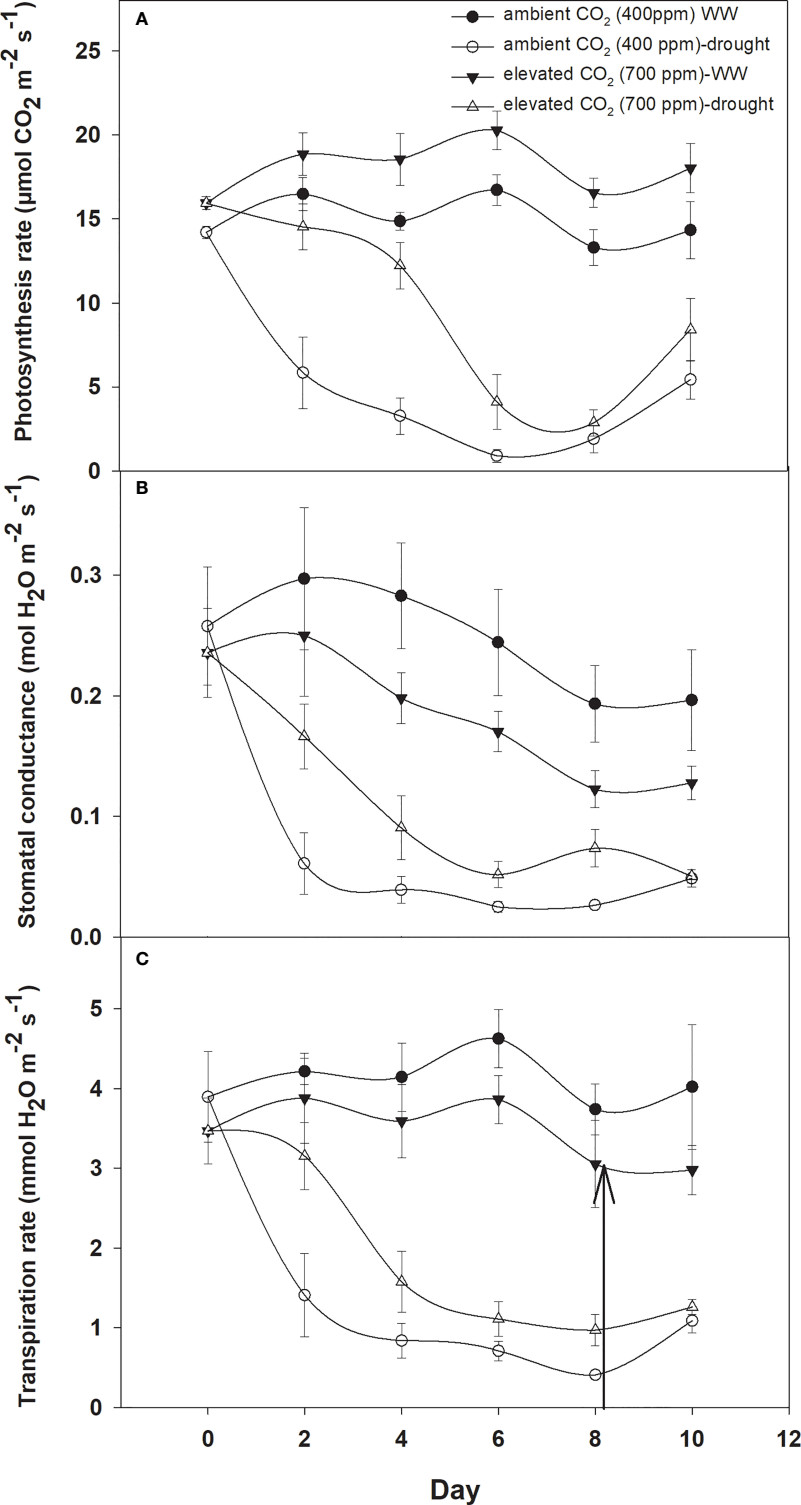
Effect of CO_2_ concentrations and moisture conditions on **(A)** photosynthesis rate, **(B)** stomatal conductance, and **(C)** transpiration rate, of *Datura stramonium*. The vertical line on the data point represents the standard error of the mean and the vertical arrow line after Day 8 represents the addition of water in the drought treatments. WW, well-watered.

Photosynthesis activities were recorded for *D. stramonium* under two moisture conditions (well-watered and drought) at 400 ppm and 700 ppm CO_2_ concentrations (**Figure 1A**). These measurements were recorded for 10 days at two-day intervals. It is depicted in **Figure 1A** that elevated CO_2_ increased the photosynthesis rate by 2–3 µmol CO_2_ m^−2^ s^−1^ when compared to that with ambient CO_2_. However, the photosynthesis rate was variable under drought conditions with the two CO_2_ concentrations. Under drought conditions, the photosynthesis rate under aCO_2_ decreased from 14 to 1 µmol CO_2_ m^−2^ s^−1^ at Day 8, while with eCO_2_ this reduction was less, being 2.8 µmol CO_2_ m^−2^ s^−1^ photosynthesis rate at Day 8. This clearly indicates that eCO_2_ mitigated the adverse effects of drought on *D. stramonium*. After the measurement, water was added to the drought-treated plants to investigate the recovery response. The photosynthesis rate was seen to recover, showing a higher rate with eCO_2_ compared to that under aCO_2_. **Figure 1** showed that the photosynthesis rate increased from 2.86 to 8.4 µmol CO_2_ m^−2^ s^−1^ with eCO_2_, while the corresponding recovery under aCO_2_ was 5.4 µmol CO_2_ m^−2^ s^−1^. The ANOVA table indicated that CO_2_ (p<0.001), time (p<0.001), water (p<0.001), CO_2_ × time (p<0.001), and time × water (p<0.001) interactions were significant for photosynthesis rate, while the interactions of CO_2_ × water and CO_2_ × time × water were non-significant for this parameter.

When *D. stramonium* was grown under well-watered conditions, the stomatal conductance was higher with aCO_2_ compared to that with eCO_2_ (**Figure 1B**). Moreover, the stomatal conductance under drought conditions showed variable responses to both CO_2_ concentrations (400 ppm and 700 ppm). The decline in stomatal conductance due to drought conditions was higher with aCO_2_ and reached 0.03 mol H_2_O m^−2^ s^−1^ on Day 8. Stomatal conductance with eCO_2_ on Day 8 was 0.07 mol H_2_O m^−2^ s^−1^. Re-watering the *D. stramonium* sample after the Day 8 measurement did not significantly improve the stomatal conductance and recorded only 0.05 mol H_2_O m^−2^ s^−1^ conductance. The ANOVA results in **Table 1** showed that time (p<0.001), water (p<0.001), CO_2_ × water (p<0.001), and time × water (p<0.001) were all significant for stomatal conductance. The rest of the effects were non-significant for this parameter.

**Table 1:** Summary of the analysis variance for physiological parameters of *Datura stramonium* in response to CO_2_ levels, water condition and time of observation.

Parameter	CO_2_		Time		Water		CO_2_ × Time		CO_2_ × Water		Time × Water		CO_2_×Time×Water	
	*p*-value	HSD at 0.05	*p*-value	HSD at 0.05	*p*-value	HSD at 0.05	*p*-value	HSD at 0.05	*p*-value	HSD at 0.05	*p*-value	HSD at 0.05	*p*-value	HSD at 0.05
Photosynthesis rate µmol CO_2_ m^−2^ s^−1^	0.000	0.95	0.000	2.42	0.000	0.95	0.040	3.94	0.158	1.77	0.000	3.94	0.057	6.23
Stomatal conductance mol H_2_O m^−2^ s^−1^	0.312	0.03	0.000	0.06	0.000	0.03	0.788	0.10	0.000	0.47	0.001	0.10	0.579	0.17
Transpiration rate mmol H_2_O m^−2^ s^−1^	0.752	0.32	0.000	0.82	0.000	0.32	0.352	1.33	0.000	0.60	0.000	1.33	0.629	2.11
Intercullular CO_2_ concentration µmol mol^−1^	0.000	19.87	0.005	50.44	0.711	19.87	0.165	82.14	0.140	37.03	0.008	82.14	0.105	129.86
Water use efficiency mmol CO_2_ mol^−1^ H_2_O	0.001	0.93	0.110	2.37	0.024	0.93	0.322	3.86	0.580	1.74	0.216	3.86	0.172	6.10
Intrinsic water use efficiency mmol CO_2_ mol^−1^ H_2_O	0.000	16.88	0.000	42.86	0.000	16.88	0.107	69.76	0.006	31.45	0.000	69.76	0.240	110.28
Instantaneous carboxylation efficiency µmolm^−2^ s^−1^ Pa^−1^	0.000	0.04	0.000	0.01	0.917	0.04	0.150	0.02	0.433	0.07	0.000	0.02	0.330	0.03
Minimum fluorescence (FÓ)	0.106	16.60	0.093	42.13	0.522	16.60	0.033	68.60	0.339	30.93	0.126	68.60	0.362	108.45
Maximum fluorescence (F́)	0.000	30.84	0.000	78.27	0.000	30.84	0.000	127.46	0.280	57.46	0.000	127.46	0.977	201.50
Quantum yield of PSII	0.970	0.02	0.000	0.05	0.000	0.02	0.060	0.07	0.080	0.03	0.000	0.07	0.419	0.11
Photochemical efficiency of PSII (Fv́/Fḿ)	0.000	0.02	0.000	0.02	0.000	0.02	0.031	0.09	0.400	0.04	0.001	0.09	0.040	0.14
Photochemical quenching (qP)	0.148	0.03	0.000	0.08	0.000	0.03	0.422	1.33	0.167	0.06	0.000	1.33	0.676	0.21
Photosynthetic electron transport rate µmole^−1^ m^−2^ s^−1^	0.980	7.76	0.000	19.23	0.000	7.76	0.626	31.32	0.822	14.12	0.000	31.32	0.413	49.51

CO_2_ was 400 and 700 ppm; water condition was 100 and 60% field capacity; time of observations was 0 to 10 days at two days intervals from the start of drought conditions.

*D. stramonium* exhibited a significant difference in transpiration rate under eCO_2_ and aCO_2_ when grown under well-watered or drought conditions (**Figure 1C**). Elevated CO_2_ reduced the transpiration rate compared to that with aCO_2_ under well-watered conditions. Under this water regime, transpiration rate with aCO_2_ was recorded up to 4.6 mmol H_2_O m^−2^ s^−1^ while it was 3.8 mmol H_2_O m^−2^ s^−1^ with eCO_2_. Drought stress resulted in a progressive decline in transpiration rate under both concentrations of CO_2_ until Day 8, but this decline was significantly less with eCO_2_. The transpiration rate was 0.97 mmol H_2_O m^−2^ s^−1^ at Day 8 with eCO_2,_ whereas it was 0.41 mmol H_2_O m^−2^ s^−1^ with aCO_2_. Moreover, recovery in transpiration was higher with eCO_2_ upon re-instating the water to drought treatments (**Figure 1C**). The main effects of time (p<0.001) and water (p<0.001) and the interaction of CO_2_ × water (p<0.001) and time × water (p<0.001) were significant for the transpiration rate.

**Figure 2:**
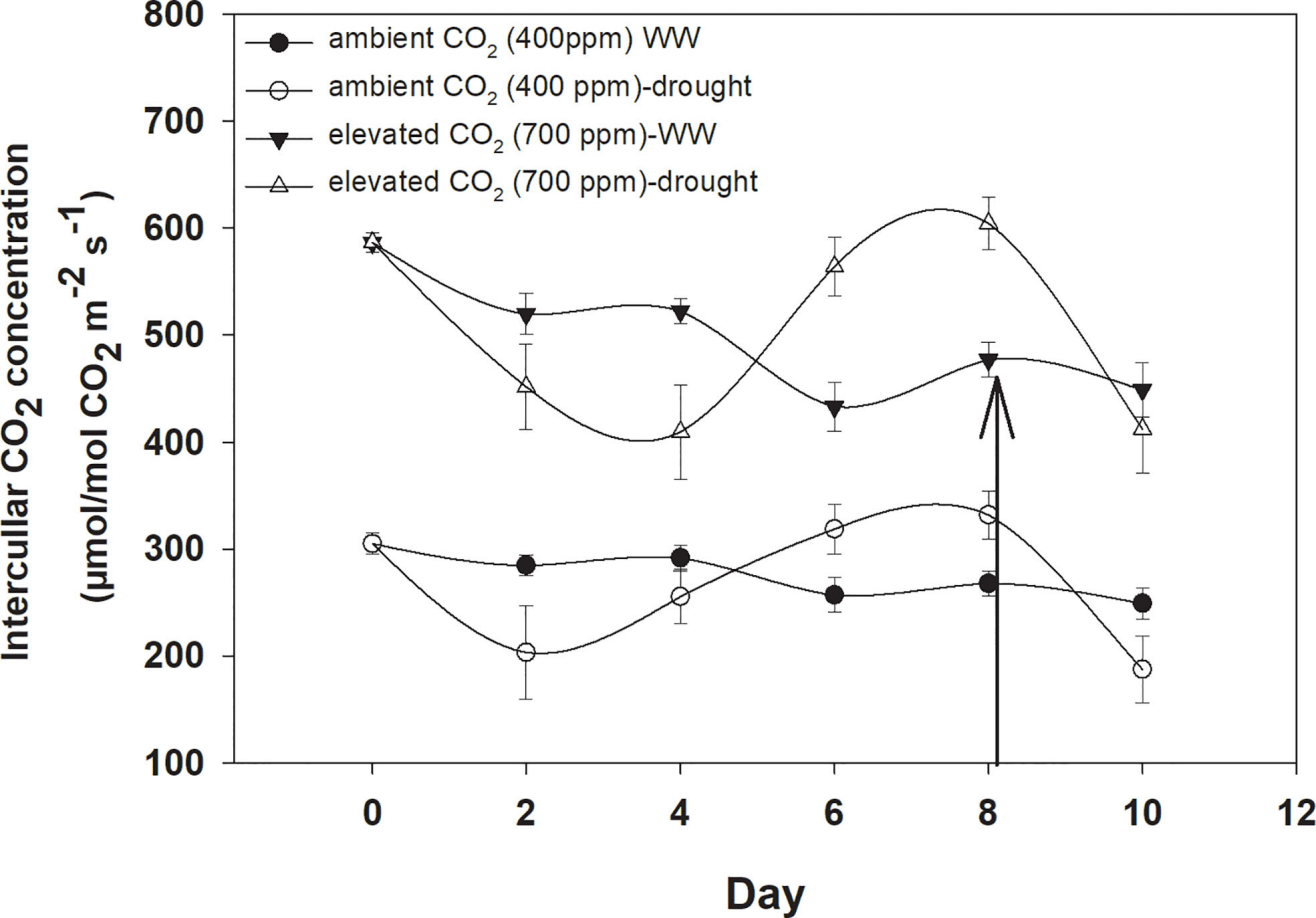
Effect of CO_2_ concentrations and moisture conditions on intercellular CO_2_ concentration of *Datura stramonium*. The vertical line on the data point represents the standard error of the mean and the vertical arrow line after Day 8 represents the addition of water in the drought treatments. WW, well-watered.

Elevated CO_2_ concentration increased the intercellular CO_2_ concentration compared to that with aCO_2_ (**Figure 2**). *D. stramonium* grown under well-water conditions recorded about 430–586 µmol mol^−1^ intercellular CO_2_ concentration with aCO_2_ compared with eCO_2_, where it was 249–304 µmol mol^−1^. Drought conditions were seen to have drastic effects on intercellular CO_2_ concentration, and this was increased with an increase in drought duration. The ANOVA values in **Table 1** showed that the main effects (CO_2_ and time) and interaction of time × water were significant for intercellular CO_2_ concentration (p<0.009).

### Water use efficiency

**Figure 3:**
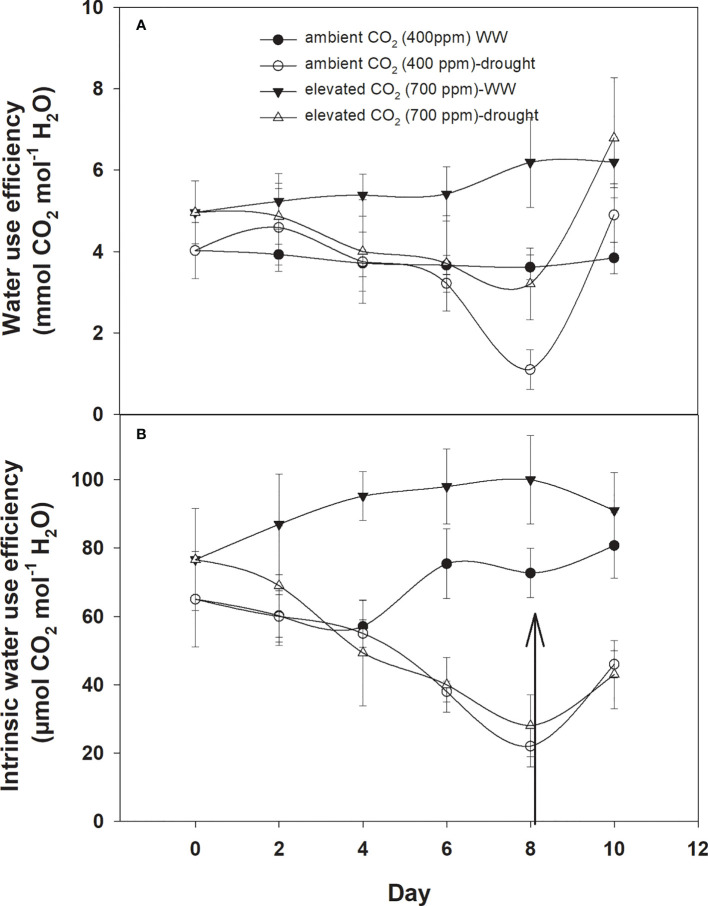
Effect of CO_2_ concentrations and moisture conditions on **(A)** water use efficiency and **(B)** intrinsic water use efficiency of *Datura stramonium*. The vertical line on the data point represents the standard error of the mean and the vertical arrow line after Day 8 represents the addition of water in the drought treatments. WW, well-watered.

Under well-watered conditions, water use efficiency was 5–6 with eCO_2_ compared to that of aCO_2_, which recorded 3–4 mmol CO_2_ mol^−1^ H_2_O (**Figure 3A**). The response of *D. stramonium* towards water use efficiency under drought conditions was variable, with water use efficiency decreasing with an increase in drought duration until Day 8. The decline in water use efficiency with eCO_2_, was lower than that with aCO_2_. Water use efficiency was reduced from 4 to 1.6 mmol CO_2_ per mol of water under eCO_2_ whereas this decline was up to 2.1 mmol CO_2_ per mol of water with aCO_2_ concentration under drought conditions. The re-instatement of water in drought treatment at Day 8 led to a significant recovery in water use efficiency, reaching 4.8 mmol CO_2_ mol^−1^ H_2_O in the treatment where a 400 ppm CO_2_ concentration was maintained. By comparison, water use efficiency reached 5.7 mmol CO_2_ mol^−1^ H_2_O when the CO_2_ concentration was maintained at 700 ppm. Statistical analysis of water use efficiency in **Table 1** showed that the mean effects of CO_2_ (p<0.001) and water (p<0.024) were significant, whereas the main effects of time and all interactions were non-significant.

Similar to water use efficiency, the intrinsic water use efficiency of *D. stramonium* was higher with eCO_2_ compared to that with aCO_2_ under well-watered conditions (**Figure 3B**), while the intrinsic water use efficiency under drought conditions with aCO_2_ or eCO_2_ was reduced at variable rates. A progressive decline in intrinsic water use efficiency under eCO_2_ in drought treatment conditions started at Day 6 and reached a minimum (49 mmol CO_2_ per mol of water) at Day 8. With aCO_2_ in drought conditions, the progressive decline started on Day 4 and reached 31 mmol CO_2_ per mol of water on Day 8. The addition of water at this date significantly recovered intrinsic water use efficiency. Elevated CO_2_ and aCO_2_ resulted in 162.2 and 110.5 mmol CO_2_ per mol of water as their intrinsic water use efficiency, respectively. Data from ANOVA showed that the main effects of CO_2_ (p<0.001), time (p<0.001), and water (p<0.0001), plus interaction of CO_2_ × water (p = 0.006), and time × water (p<0.001), were significant for the intrinsic water use efficiency of *D. stramonium*. The three-way interaction was non-significant for this parameter.

### Carboxylation efficiency

**Figure 4:**
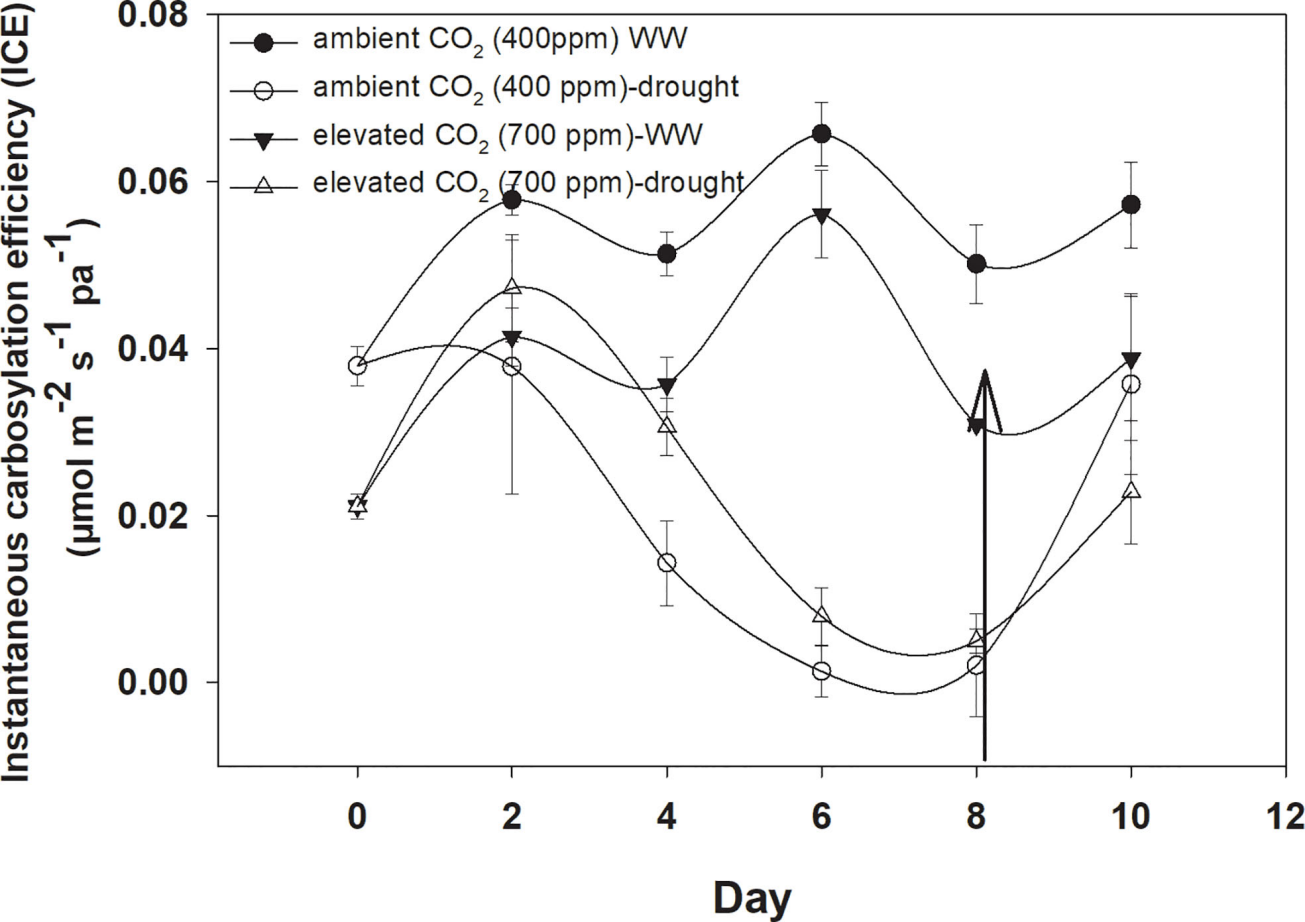
Effect of CO_2_ concentrations and moisture conditions on instantaneous carboxylation efficiency of *Datura stramonium*. The vertical line on the data point represents the standard error of the mean and the vertical arrow line after Day 8 represents the addition of water in the drought treatments. WW, well-watered.

**Figure 5:**
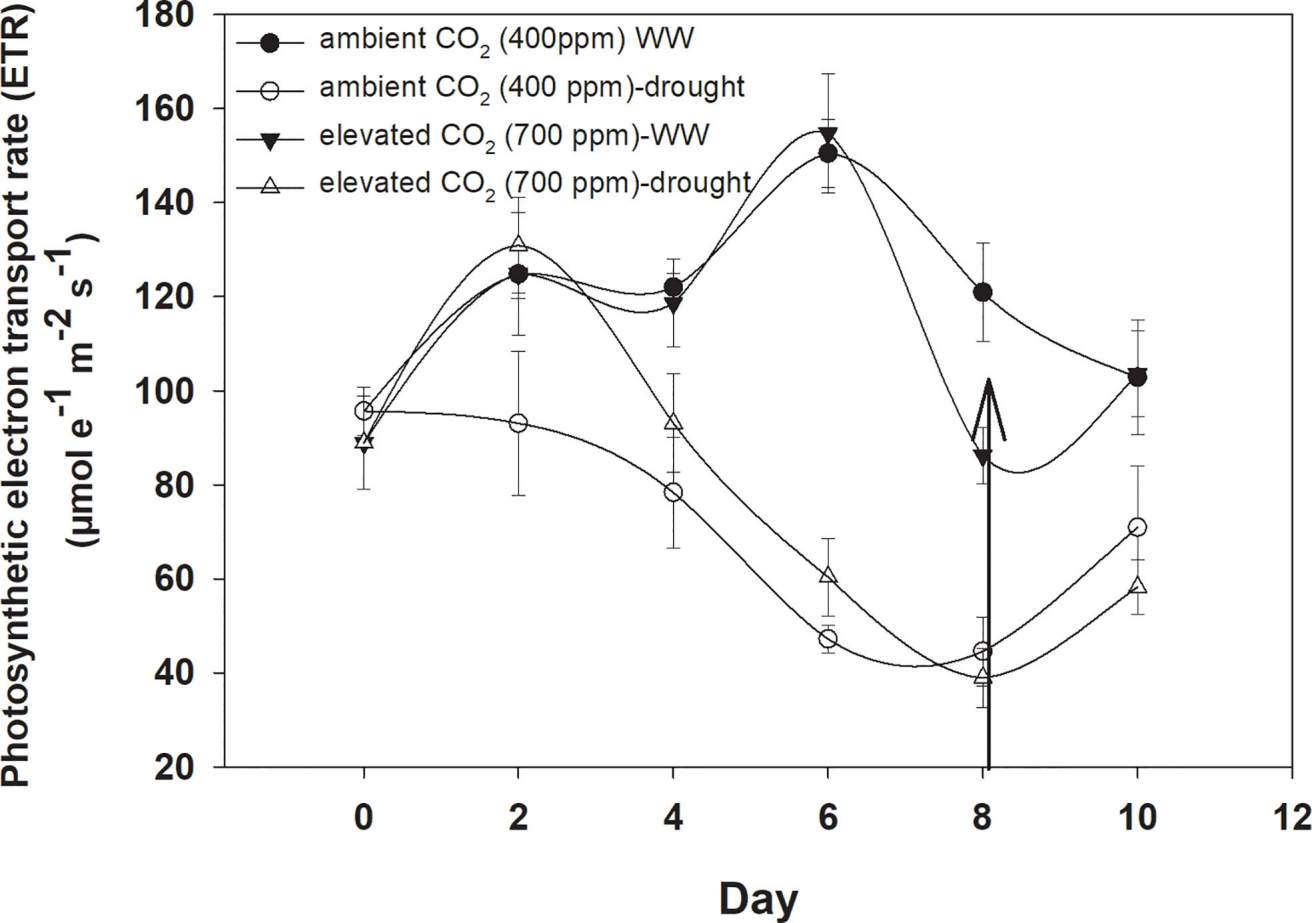
Effect of CO_2_ concentrations and moisture conditions on the photosynthetic electron transport rate of *Datura stramonium*. The vertical line on the data point represents the standard error of the mean and the vertical arrow line after Day 8 represents the addition of water in the drought treatments. WW, well-watered.

Under well-watered conditions, elevated CO_2_ caused a significant reduction in instantaneous carboxylation efficiency when compared with aCO_2_ (**Figure 5**). The instantaneous carboxylation efficiency values were between 0.05 and 0.07 µmol m^−2^ s^−1^ Pa^−1^ under eCO_2,_ whereas it was 0.03–0.04 µmol m^−2^ s^−1^ Pa^−1^ under aCO_2_. Drought conditions resulted in a variable response for instantaneous carboxylation efficiency for *D. stramonium*. Drought caused reductions in instantaneous carboxylation efficiency with an increase in the duration of drought, but this decline was much more progressive under aCO_2_ compared to that with eCO_2_. **Figure 4** shows that the instantaneous carboxylation efficiency decline started at the second day of drought stress, compared with the decline starting at Day 4 with eCO_2_ under drought conditions. The addition of water at Day 8 to drought treatments increased the instantaneous carboxylation efficiency under both CO_2_ concentrations but instantaneous carboxylation efficiency reached 0.04 µmol m^−2^ s^−1^ Pa^−1^ with aCO_2_ after water addition to the drought treatment. The ANOVA results presented in **Table 1** showed that the main effects of CO_2_ (p<0.001), time (p<0.000), water (p<0.000), and interaction of time × water (p<0.000) were significant for instantaneous carboxylation efficiency of *D. stramonium*, whereas the rest of the interactions were non-significant.

### Photosynthetic electron transport

Under well-watered conditions, the photosynthetic electron transport rate was higher with an eCO_2_ concentration than with aCO_2_ (**Figure 5**). Drought significantly affected the photosynthetic electron transport efficiency with every increment in the drought days and the photosynthetic electron transport rate reached a minimum value (44 µmol e^−1^ m^−2^ s^−1^ for aCO_2_ and 38 µmol e^−1^ m^−2^ s^−1^ for eCO_2_) on Day 8 of measurement under both CO_2_ concentrations. Water addition to drought treatments recovered the photosynthetic electron transport rate under both CO_2_ concentrations. The photosynthetic electron transport rate of *D. stramonium* was significant in response to time and water, and the interaction time × water. The rest of the interactions were seen to be non-significant (**Table 1**).

### Fluorescence

**Figure 6:**
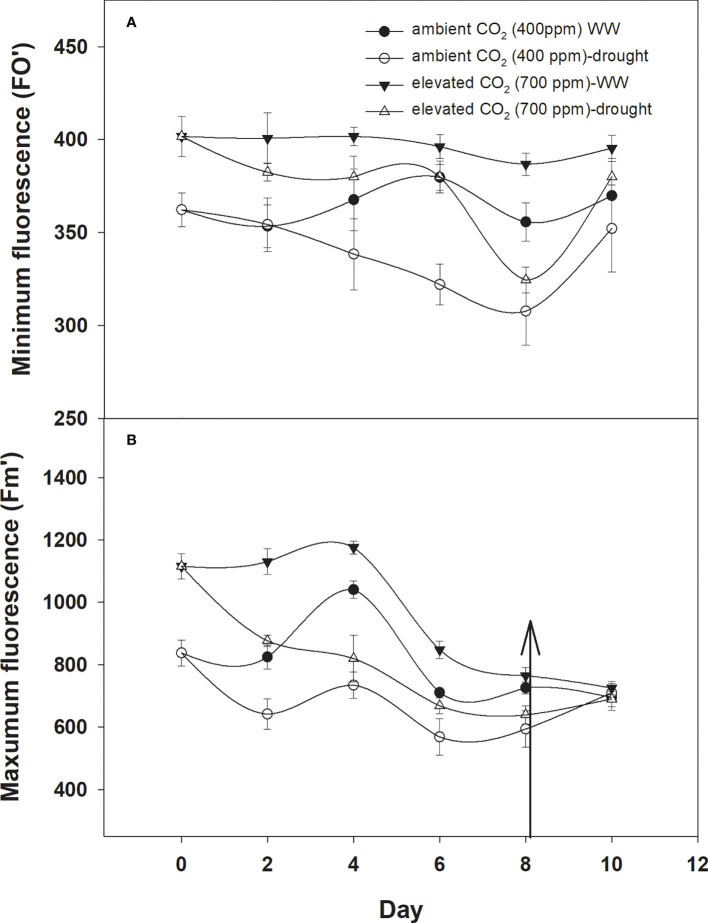
Effect of CO_2_ concentrations and moisture conditions on **(A)** minimum fluorescence and **(B)** maximum fluorescence of *Datura stramonium*. The vertical line on the data point represents the standard error of the mean and the vertical arrow line after Day 8 represents the addition of water in the drought treatments. WW, well-watered.

**Figure 7:**
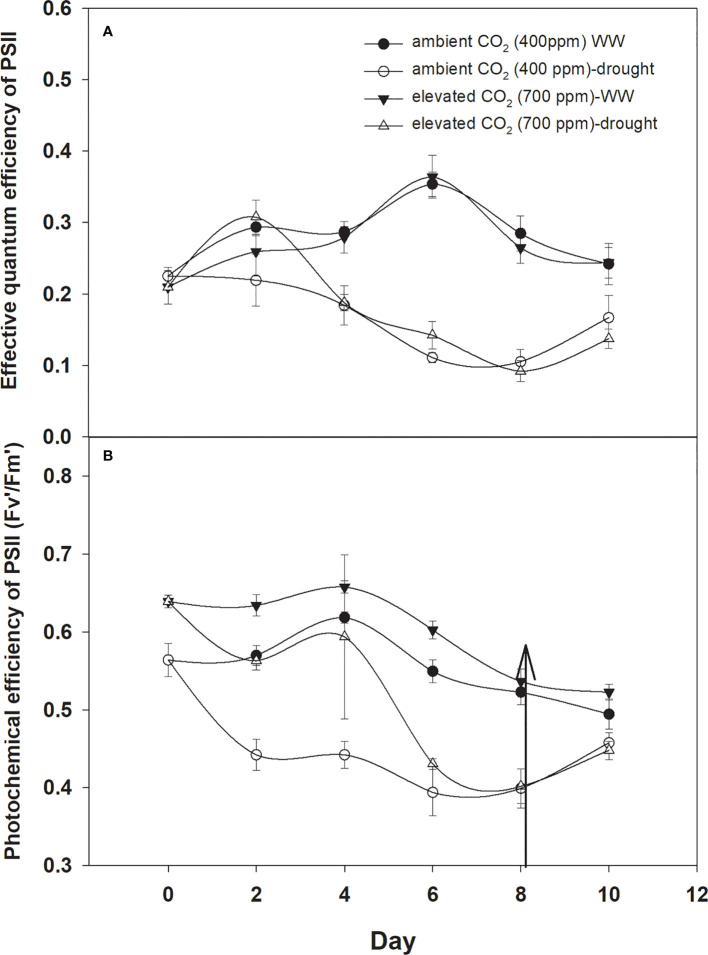
Effect of CO_2_ concentrations and moisture conditions on **(A)** effective quantum efficiency of PSII and **(B)** photochemical efficiency of PSII of *Datura stramonium*. The vertical line on the data point represents the standard error of the mean and the vertical arrow line after Day 8 represents the addition of water in the drought treatments. WW, well-watered.

The minimum and maximum fluorescence values of *D. stramonium* grown in well-watered conditions were higher with eCO_2_ compared to with aCO_2_ (**Figures 7A, B**). The effect of drought was more pronounced on minimum fluorescence under aCO_2_ as the minimum fluorescence decreased linearly until Day 8 of observation with an increase in drought duration. By comparison, eCO_2_ under drought conditions had no effect on minimum fluorescence until Day 6, and after that, it declined to 319 (**Figure 6A**). Maximum fluorescence under drought conditions was higher with eCO_2_ compared to that with aCO_2_, and a decline in maximum fluorescence occurred with both CO_2_ concentrations with an increase in drought conditions until Day 8 of the observation (**Figure 6B**). Water addition to drought treatment significantly recovered the minimum fluorescence under both CO_2_ concentrations, while water addition to drought treatments slightly recovered the maximum fluorescence. The ANOVA table results showed that the interaction of CO_2_ × time (p = 0.03) was significant for the minimum fluorescence of *D. stramonium* while the effects of CO_2_ (p<0.001), time (p<0.001), water (p<0.001), and the interaction of CO_2_ × time (p<0.001), and time × water (p<0.001) were significant for the maximum fluorescence of *D. stramonium.*

### PSII activity

The effective quantum efficiency of PSII with both CO_2_ concentrations was similar when *D. stramonium* was grown under well-watered conditions (**Figure 7A**). However, drought conditions showed the variable response of the effective quantum efficiency of PSII under tested CO_2_ concentrations. Elevated CO_2_ concentration slightly mitigated the adverse effects of drought, and the effective quantum efficiency of PSII was sustained up to Day 4 with an increase in drought duration. By comparison, aCO_2_ linearly decreased the effective quantum efficiency of PSII with an increase in drought duration until Day 8. Addition of water to drought treatments recovered the effective quantum efficiency of PSII of *D. stramonium* in the same way under both CO_2_ concentrations. The main effects of time (p<0.001), water (p<0.001), and interaction of time × water (p<0.001) were non-significant for the effective quantum efficiency of PSII.

The photochemical efficiency of PSII was significantly higher with eCO_2_ compared to that with aCO_2_ (**Figure 7B**). Drought had significant effects on this parameter and effects were more pronounced on the photochemical efficiency of PSII under aCO_2,_ significantly decreasing with the initiation of drought and declining from 0.56 to 0.40 from drought Day 1 to Day 8. On the other hand, drought effects were slight on the photochemical efficiency of PSII until Day 4 of the drought, and after that, it declined to 0.40 at Day 8 of the drought duration. Water addition to drought treatment grown under both CO_2_ concentrations recovered the photochemical efficiency of PSII in the same way (**Figure 7**). Results of the ANOVA showed that time (p<0.001), water (p<0.001), CO_2_ (p<0.001), and time × water (p = 0.001) have significant effects on the photochemical efficiency of PSII (**Table 1**).

### Photochemical quenching

**Figure 8:**
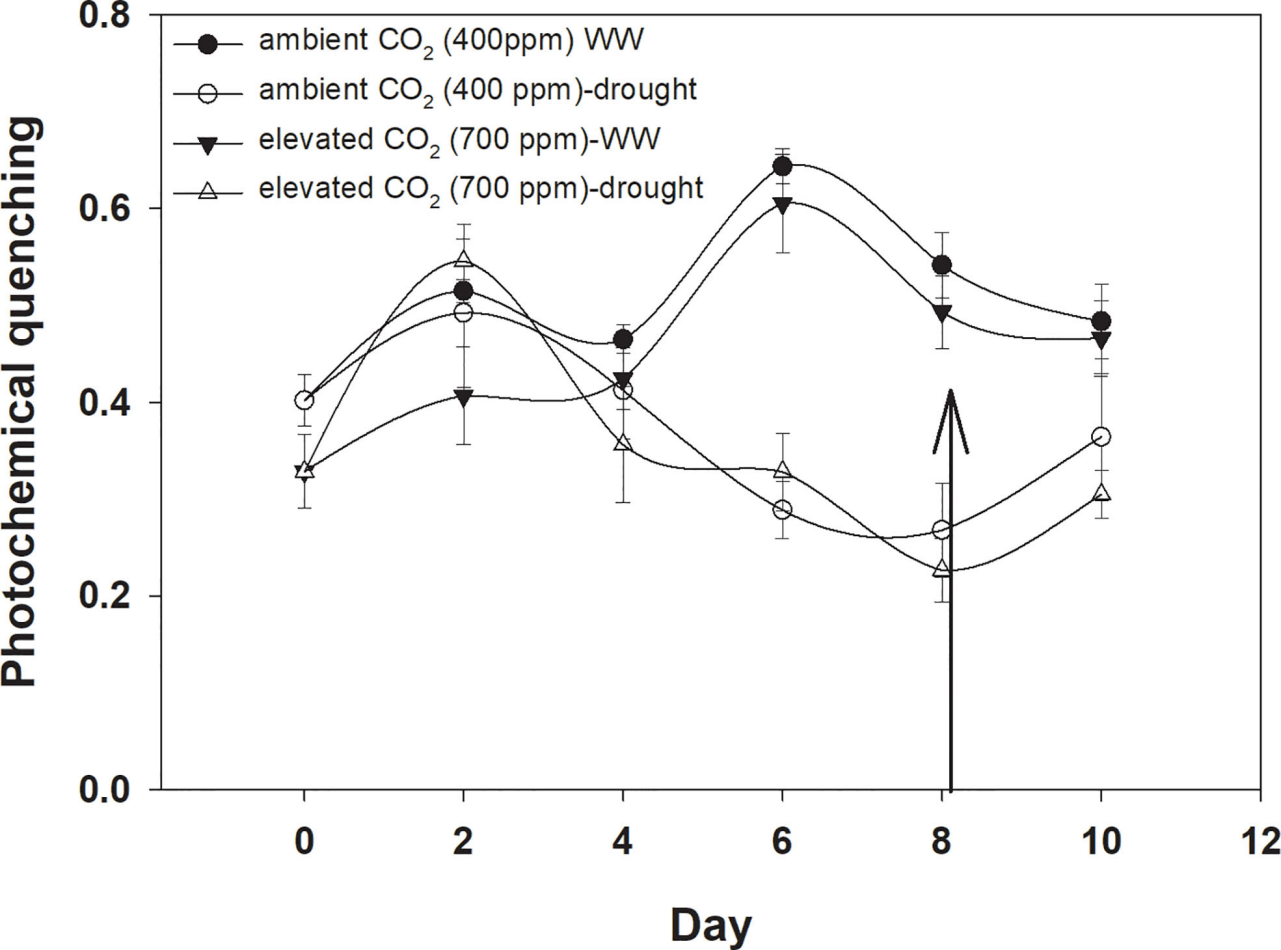
Effect of CO_2_ concentrations and moisture conditions on photochemical quenching of *Datura stramonium*. A nail on the data point represents the standard error of the mean and the vertical arrow line after Day 8 represents the addition of water in the drought treatments. WW, well-watered.

Photochemical quenching was higher with aCO_2_ compared to that with eCO_2_ when *D. stramonium* was grown under well-watered conditions (**Figure 8**). Drought stress significantly reduced photochemical quenching with an increase in drought duration, and this decline was in a similar way under both CO_2_ concentrations. The addition of water to drought treatments on Day 8 recovered photochemical quenching, with this recovery being higher with aCO_2_. The ANOVA results indicated that the effects of time (p<0.001), water (p<0.001), and time × water (p<0.001) were significant for photochemical quenching of *D. stramonium* (**Table 1**).

## Discussion

Climate change has led to significant variation in rainfall patterns, and this, together with increased atmospheric CO_2_ concentrations, will increase wheat-crop competition for available resources (Ramesh et al., 2017; Bajwa et al., 2019; Chadha et al., 2020). This study revealed that atmospheric CO_2_ concentrations (400 ppm and 700 ppm) and limited water conditions had variable effects on gas exchange parameters, photosynthetic electron transport rate, and water use efficiency of *D. stramonium*. Drought conditions had negative effects on the photosynthesis rate of *D. stramonium*, but elevated CO_2_ concentrations mitigated the adverse effects of drought on the photosynthesis rate of *D. stramonium*. Moreover, eCO_2_ increased the photosynthetic rate under normal irrigation conditions. It is well documented that drought causes a range of responses in photosynthesis mechanisms, depending upon species and season (Alba et al., 2019; Dusenge et al., 2019). Ji et al. (2015) reported that eCO_2_ increased the leaf photosynthetic rate. In our study under well-watered conditions, the photosynthesis rate was increased by about 25% with eCO_2_ compared to that of ambient CO_2_ concentration. Similarly, eCO_2_ also mitigated the adverse effects of drought and increased the net photosynthesis rate by 12% under drought conditions, and the net photosynthesis rate was 50% higher with elevated CO_2_ under severe drought conditions. Furthermore, it is generally believed that photosynthesis increases with eCO_2,_ even under stressful environments (Ghahramani et al., 2019; De Kauwe et al., 2021). *D. stramonium* grown under eCO_2_ exhibited lower stomatal conductance compared to that which was grown under aCO_2_. According to Habermann et al. (2019), plants grown under eCO_2_ in drought conditions resulted in greater stomatal closure than those grown under aCO_2_. Stomatal conductance is considered to decrease both photosynthesis rate and CO2 concentration in intercellular spaces of the leaf which inhibits metabolism (Cornic, 2000). However, the relative importance of stomatal conductance in restricting the supply of CO_2_ to metabolism (stomatal limitation) and impairment, which decreases the potential rate, of photosynthesis rate is unclear. Moreover, metabolic limitation is often observed and correlates with loss of ATP, which stands to decrease with mild water loss (Lawlor, 2002). In our study, elevated CO_2_ maintained stomatal functioning under water stress.

The study by Khalid et al. (2019) found that the stomatal conductors of *D. alba* decreased due to an increase in CO_2_ concentration along roadsides. Our results showed a progressive decline in transpiration rate under both CO_2_ concentrations, but this decline was significantly less with eCO_2_. The decline in transpiration rate might be due to stomatal closure, stimulated by a combination of eCO_2_ and drought stress. A study by Walia et al. (2022) showed that elevated CO_2_ decreased stomatal and transpiration rates by partial closure of stomata, resulting in increased water use efficiency and lower water stress in the plant. Robredo et al. (2007) found that plants grown under eCO_2_ have more water use efficiency and developed water stress more slowly than those grown under ambient CO_2_ concentration. Moreover, eCO_2_ reduced the drought-induced damage in *D. stramonium*, with plant recovery being significant upon re-commencement of water and drought condition treatments. In previous work, it was noted that the degree of recovery depends upon species and drought duration (Duan et al., 2022). Robredo et al. (2007) also noted that barley plants were not able to recover stomatal closure upon re-watering after 16 days of drought stress. In our study, intercellular CO_2_ concentration increased with an increase in CO_2_ concentration, and also that drought stress has drastic effects on intercellular CO_2_, which increases with an increase in drought duration. Our findings are in line with those of Wang et al. (2018) and Dregulo (2022), who reported that stomatal closure and reduced transpiration rate were found under an enriched CO_2_ environment. In another study, it was clearly shown that limitations on photosynthesis rate due to drought stress are based on the significant decrease in stomatal conductance and intercellular concentration (Salmon et al., 2020).

The water use efficiency of *D. stramonium* was 40% higher with eCO_2_ compared to that with aCO_2_ under well-watered conditions, suggesting that eCO_2_ mitigates the adverse effects of drought on the water use efficiency of *D. stramonium*. This might be due to the reduced transpiration rate as stomatal closure is seen under eCO_2_. Indeed, as is broadly understood from our results together with other research, water use efficiency enhancement is the result of reduced stomatal conductance and enhanced net photosynthesis rate under a CO_2_-enriched environmental context (Wei et al., 2022; Wu et al., 2022). Similar to water use efficiency, the intrinsic water use efficiency of *D. stramonium* shows similar trends under eCO_2_ and drought conditions. Soil moisture conservation of many species has been reported when they are grown under elevated CO_2_ concentrations. Our results are further supported by Hassan et al. (2022), who reported that eCO_2_ reduced the impact of limited water conditions on the physiological traits of a number of plants. Drought conditions caused a reduction in instantaneous carboxylation efficiency with an increase in the duration of drought, but this decline was much lower with eCO_2_ when compared to that of aCO_2_. Our findings are in line with those of Wang et al. (2015) and Pérez-López et al. (2013), who showed a significant decline in instantaneous carboxylation efficiency with eCO_2_. Under well-watered conditions, photosynthetic electron transport rate was higher with aCO_2,_ whereas photosynthetic electron transport rate decreased with the length of drought duration. This suggests that a higher rate of electron transport occurs in drought-stress environments coupled with elevated CO_2_ concentrations. These results are in line with the studies of the responses of Japanese white birch (*Betula platyphylla* **var.** *japonica*) to elevated carbon dioxide and drought, where photosynthetic electron transport rate was decreased by elevated CO_2_ but was elevated under drought in ambient CO_2_ conditions (Kitao et al., 2007). The minimum and maximum fluorescence values of *D. stramonium* grown in well-watered conditions were higher with eCO_2_ compared to those with aCO_2_. The results are in line with those of Chadha et al. (2020) and Weller et al. (2021), who suggested that fluorescence increased with an increase in atmospheric CO_2_.

The effective quantum efficiency of PSII was decreased in *D. stramonium* under drought in both CO_2_ treatments. However, elevated CO_2_ slightly mitigated the adverse effects on the effective quantum efficiency of PSII. Such trends have been observed in previous studies (Perveen et al., 2010; Wang et al., 2018), where it was concluded that elevated CO_2_ and drought decreased the effective quantum efficiency of PSII. However, the photochemical efficiency of PSII of *D. stramomium* was slightly increased with elevated CO_2_, indicating that a CO_2_-enriched environment reduces the risk of damage to PSII by abiotic stress. Similarly, Zhao et al. (2010) showed an increase in the photochemical efficiency of PSII in *Betula platyphylla* seedlings under 700 ppm CO_2_.

In conclusion, drought affects different physiological traits of *D. stramonium*. Nevertheless, eCO_2_ generally increased the leaf gas exchange parameters, water use efficiency, and PSII activity of *D. stramonium*, by comparison with ambient CO_2_. This would have contributed to maintaining a higher leaf water potential, so the improved water status of drought-treated plants at elevated CO_2_. As a result, plants exposed to high CO_2_ increased their net photosynthesis rate, which, when coupled with reduced stomatal closure and transpiration rate, led to increased water use efficiency. It therefore appears that when *D. stramonium* grows under elevated CO_2_ conditions, this mitigates or delays the effects of water stress. Therefore, under the anticipated changes in drought and rainfall patterns, this species may be found to be more competitive, thereby increasing its invasive range, which will result in its having a more serious agricultural impact in the future.

## Data availability statement

The raw data supporting the conclusions of this article will be made available by the authors, without undue reservation.

## Author contributions

SF conceived and designed the study. MJ conducted the experiments. All authors were involved in writing and reviewing the manuscript. All authors contributed to the article and approved the submitted version.

## Conflict of interest

JM was employed by companies Twój Swiat Jacek Mojski and Fundacja Zielona Infrastruktura.

The remaining authors declare that the research was conducted in the absence of any commercial or financial relationships that could be construed as a potential conflict of interest.

## Publisher’s note

All claims expressed in this article are solely those of the authors and do not necessarily represent those of their affiliated organizations, or those of the publisher, the editors and the reviewers. Any product that may be evaluated in this article, or claim that may be made by its manufacturer, is not guaranteed or endorsed by the publisher.
