# Supplementary figures and images for: Interactive effect of elevated CO2 and drought on physiological traits of Datura stramonium
Source: Front Plant Sci. 2022 Oct 26;13:929378. doi: 10.3389/fpls.2022.929378 (PMC9644026; doi:10.3389/fpls.2022.929378)

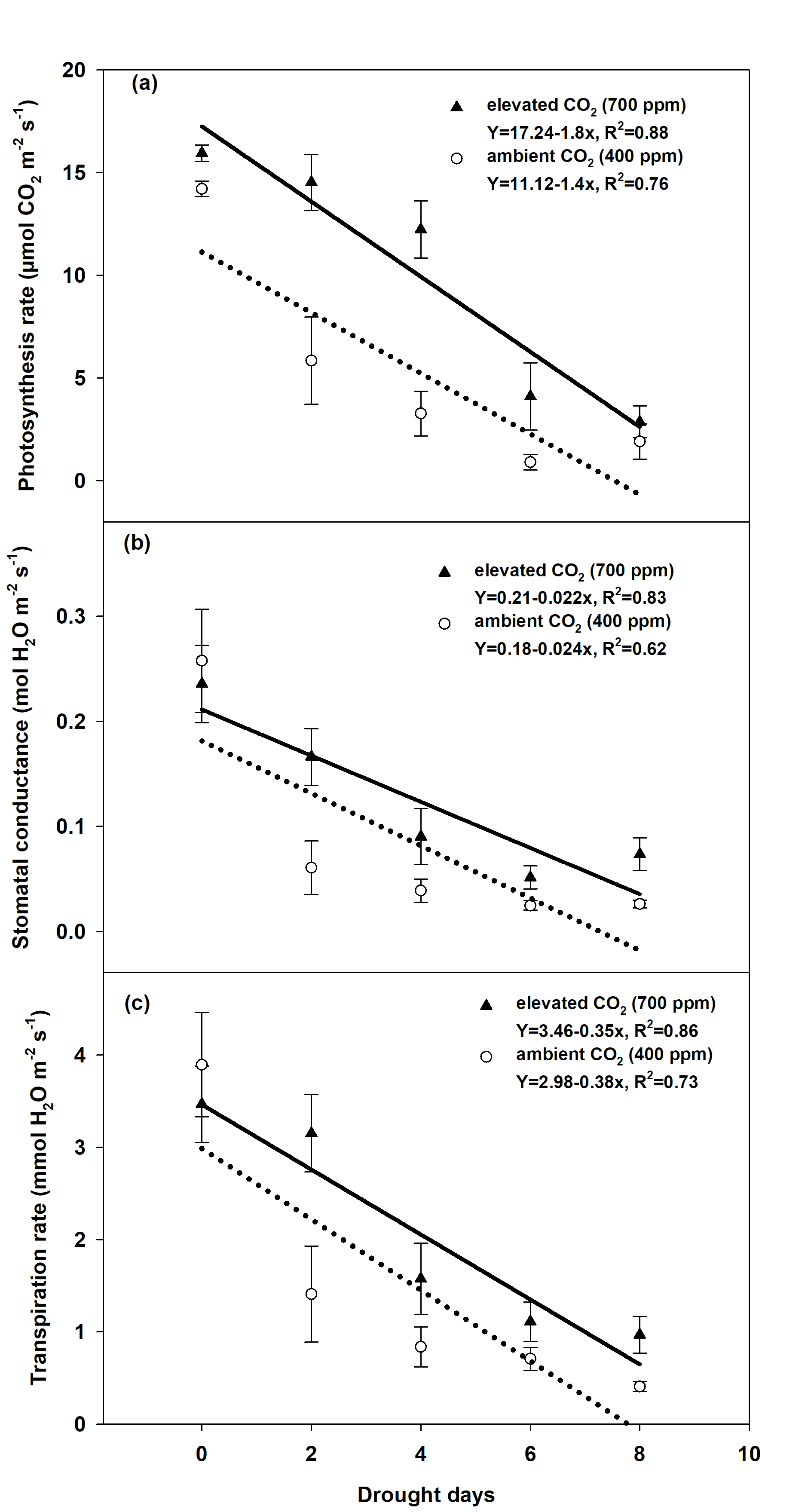

Supplement: Supplementary file 1 [file Image_1.tif]
